# Association between Vascular Endothelial Growth Factor Polymorphisms and Age-Related Macular Degeneration: An Updated Meta-Analysis

**DOI:** 10.1155/2016/8486406

**Published:** 2016-11-23

**Authors:** Martina Barchitta, Andrea Maugeri

**Affiliations:** Department of Medical and Surgical Sciences and Advanced Technologies “GF Ingrassia”, University of Catania, Catania, Via S. Sofia 87, 95123 Catania, Italy

## Abstract

Age-related macular degeneration (AMD) is the most common cause of blindness in elderly people worldwide and the major degenerative disease of the retina that leads to progressive impairment of central vision. Several polymorphisms in different genes have been proposed as factors that increase the disease susceptibility. The aim of the present study is to carry out a systematic review and an updated meta-analysis in order to summarize the current published studies and to evaluate the associations between four common vascular endothelial growth factor (*VEGF*) polymorphisms (rs833061, rs1413711, rs3025039, and rs2010963) and AMD risk, also stratifying for AMD subtypes and ethnicity. A systematic literature search in the Medline database, using PubMed, was carried out for epidemiological studies, published before June 2016. Associations of* VEGF* polymorphisms with AMD were estimated by calculating pooled odds ratios (ORs) and 95% confidence intervals (95% CIs) based on different models. Twelve articles were included in the analysis. The present meta-analysis constitutes a useful guide for readers to study AMD and adds new evidence to the growing literature on the role of* VEGF* polymorphisms in the risk of AMD. Significant associations with AMD risk were showed for rs833061, rs1413711, and rs3025039 polymorphisms but not for rs2010963.

## 1. Introduction

Age-related macular degeneration (AMD), the most common cause of blindness in elderly people worldwide [1], is the major degenerative disease of the retina that leads to progressive impairment of central vision [2]. The progression of AMD is characterized by the primary influence on debris accumulation in the early stage, followed by the accumulation of retinal pigment epithelial abnormalities in the late stage. Particularly, there are two subtypes of late AMD, distinguishable by different clinical and pathologic features. Nonexudative (dry or atrophic) AMD is characterized by the progressive loss of the retinal pigment epithelium (RPE) cell layer, resulting in the geographic atrophy (GA) of RPE and thinning of the retina. Exudative AMD is characterized by the development of choroidal neovascularization and subretinal neovascular fibrous tissue [3], resulting in the rapid deterioration in central vision [4].

Epidemiological studies suggest that the pathogenesis of this complex disorder implicates sociodemographic (age and race), environmental (cigarette smoking, light exposure, and unhealthy diet), and genetic risk factors [3, 5]. Particularly, it seems reasonable that environmental effects may be modulated by genetic factors, and environmental risk factors may trigger the disease in genetically susceptible subjects [6–8], establishing a typical gene-environment interaction [9]. Although, several polymorphisms in different genes have been proposed as factors that increase the disease susceptibility [10], but the molecular mechanisms of AMD development and progression are not completely clarified. A genome-wide association study on more than 17000 AMD patients reported that seven new genomic loci were linked to the regulation of complement activity, lipid metabolism, extracellular matrix remodelling, and angiogenesis in AMD. However, other genetic risk factors include genes encoding age-related macular degeneration 1 (*ARMD1*), apolipoprotein E (*APOE*), and complement factor H (*CFH*); vascular endothelial growth factor (*VEGF*) genes have also been reviewed [11]. VEGF has a key role in promoting angiogenesis, vasculogenesis, and lymphangiogenesis in normal and pathological cells [12]. The VEGF family molecules (placenta growth factor, VEGF-A, VEGF-B, VEGF-C, VEGF-D, and VEGF-E) are involved in development, survival, and maintenance of vessels and are essential for retinal health [13], inducing vascular leakage and inflammation [12, 14]. The human* VEGF* gene is located on chromosome 6p21.3 and contains seven introns and eight exons [15–17].

Recently, several studies have focused on the association between single-nucleotide polymorphisms (SNPs) in the* VEGF* gene and AMD risk. Among these SNPs, rs833061 (−460T/C) in the promoter region, rs1413711 (+674C/T) in intron 1, rs3025039 (+936C/T) in the 3′-untranslated region, and rs2010963 (+405G/C) in the 5′-untranslated region were found to be associated with the AMD susceptibility [18–20]. Although genetic variability of the* VEGF* gene may have a critical role in determining AMD risk, evidence by studies on small or moderate sample sizes remain ambiguous. These contentious results were also reported by recently published meta-analyses [21–23].

The aim of the present study is to carry out a more comprehensive systematic review and an updated meta-analysis in order to summarize the current published studies and to evaluate the associations between four common* VEGF* gene polymorphisms (rs833061, rs1413711, rs3025039, and rs2010963) and AMD risk, also stratifying for AMD subtypes and ethnicity.

## 2. Methods

### 2.1. Search Strategy and Selection Criteria

A systematic review of original articles, published before June 2016, was conducted by searching in the Medline database in order to collect data from epidemiological studies investigating the association between* VEGF* polymorphisms and AMD risk. The literature search, limited to studies written in English, was independently conducted by the two authors, using the following key words: (“VEGF” or “vascular endothelial growth factor”) AND (“variant” or “SNPs” or “polymorphism”) AND (“age-related macular degeneration” or “AMD”). Moreover, the references from retrieved articles were also checked to search for additional studies.

The selection criteria were as follows: (1) studies must employ a case-control or cohort design (2) and must evaluate the associations between VEGF polymorphisms (rs833061, rs1413711, rs3025039, and rs2010963) and AMD; (3) genotype data of patients and controls must be available in order to estimate odds ratios (ORs) and 95% confidence intervals (95% CIs). Furthermore, exclusion criteria were (1) studies that did not provide genotype data in AMD patients and/or in control subjects and (2) review articles. The meta-analysis was conducted according to the preferred reporting items for systematic reviews and meta-analysis (PRISMA) guidelines [24].

### 2.2. Data Extraction and Quality Assessment

The following information were extracted: first author's last name, year of publication, country and ethnicity, sample size of subjects with and without AMD, genotype distribution of case and control groups, subtypes of AMD cases and controls (dry AMD and wet AMD), and *p* value for Hardy-Weinberg equilibrium test in subjects without AMD.

### 2.3. Statistical Analyses

The REVIEW MANAGER 5.2 software, provided by the Cochrane Collaboration (http://ims.cochrane.org/revman), was used to estimate the association between VEGF polymorphisms and AMD risk.

For each polymorphism, the wild-type allele was set as 1 and the risk allele as 2 and the Chi-square test was performed to determine if the genotype distribution of the control subjects is deviated from the Hardy-Weinberg equilibrium (HWE; *p* < 0.05 was considered significant).

To calculate pooled odds ratios ORs and 95% confidence intervals (95% CIs), the following genetic models were adopted for each polymorphism: the homozygote model (22 versus 11), the dominant model (22 + 12 versus 11), the recessive model (22 versus 12 + 11), the heterozygote model (12 versus 11), and the allelic model (1 versus 2) [34].

The significance of pooled OR was determined using the *Z* test, and *p* < 0.05 was considered significant. Heterogeneity across studies was assessed using the *Q*-test based on the *χ* ^2^ statistic (*p* < 0.1 was considered statistically significant).

To quantify heterogeneity, the *I* ^2^ value was calculated and interpreted as follows: an *I* ^2^ value of 0% indicates “no heterogeneity,” whereas 25% is “low,” 50% is “moderate,” and 75% is “high” heterogeneity [35]. The between-study variance was estimated using tau-squared (*τ* ^2^) statistic [36].

According to heterogeneity across studies, the fixed-effects (Mantel-Haenszel method) or random effects models (Der Simonian-Laird method) were used to calculate pooled effect estimates.

Furthermore, subgroup analyses by subtypes of AMD and ethnicity (Asian and Caucasian) were conducted. The leave-one-out sensitivity analysis, by the omission of a single study at a time, was performed in order to assess whether a particular omission could affect the overall OR value and the heterogeneity across studies. To identify potential publication bias, the asymmetry of the funnel plots, in which ORs were plotted against their corresponding standard errors, was examined.

## 3. Results

### 3.1. Search Results and Data Characteristics

**Figure 1:**
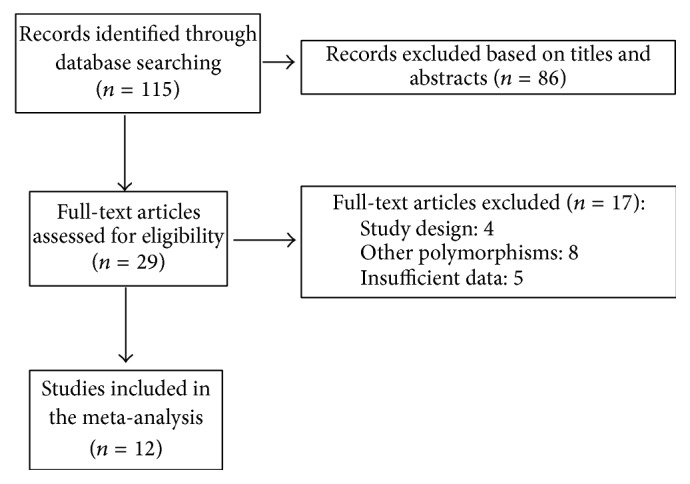
Flow diagram of study selection.

The detailed steps of the systematic review and meta-analysis process are given as a PRISMA flowchart in Figure 1. A total of 115 articles were retrieved from the database and 86 records were excluded after reading titles and/or abstracts. Thus, 29 studies were subjected to a full-text review and selected according to the selection criteria. Among these, 17 studies were identified that evaluated the association between VEGF polymorphisms and AMD risk by a case-control or cohort design [18–20, 25–33, 37–41], but five studies were excluded for insufficient data [37–41]. Consequently, 12 articles, published between 2006 and 2015, were included in the systematic review and their main characteristics are summarized in Table 1. Particularly, three studies have investigated the role of several SNPs in the AMD risk: the polymorphism rs833061 and the polymorphism rs3025039 were, respectively, analysed in six studies (rs833061: 1431 cases and 806 controls; rs3025039: 1396 cases and 1326 controls); the polymorphism rs1413711 was analyzed in four studies (554 cases and 551 controls) and the polymorphism rs2010963 in three studies (614 cases and 454 controls).

### 3.2. Meta-Analysis Results

For the rs833061 polymorphism, the meta-analysis showed a significant association with AMD under a homozygote model (CC versus TT: OR = 1.56, 95% CI 1.15–2.13); a dominant model (CT + CC versus TT: OR = 1.66, 95% CI 1.04–2.65); and an allelic model (C versus T: OR = 1.31, 95% CI 1.08–1.58). Pooled ORs, under a heterozygote model (CT versus TT: OR = 1.63, 95% CI 0.97–2.72) and a recessive model (CC versus TT + CT: OR = 1.22, 95% CI 0.94–1.59), were not statistically significant. Subgroup analysis by ethnicity confirmed that, in Asians, the polymorphism was associated with AMD under the homozygote (OR = 2.15, 95% CI 1.07–4.31), the recessive (OR = 2.04, 95% CI 1.03–4.04), and the allelic (OR = 1.28, 95% CI 1.00–1.65) models; in Caucasians, the polymorphism was associated with AMD under the homozygote (OR = 1.44, 95% CI= 1.02–2.03) and the allelic (OR = 1.33, 95% CI 1.00–1.77) models.

**Figure 2:**
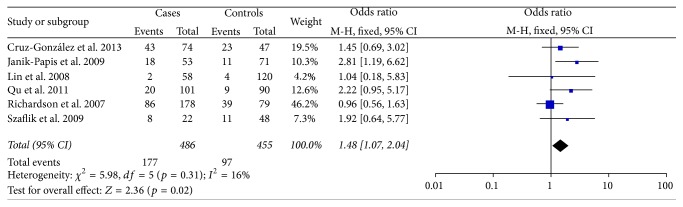
Forest plot of the association between rs833061 polymorphism and wet AMD under a homozygote model (CC versus TT).

**Figure 3:**
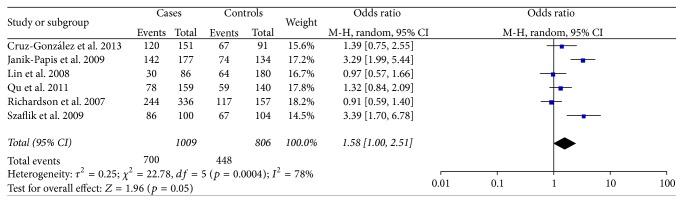
Forest plot of the association between rs833061 polymorphism and wet AMD under a dominant model (CT + CC versus TT).

**Figure 4:**
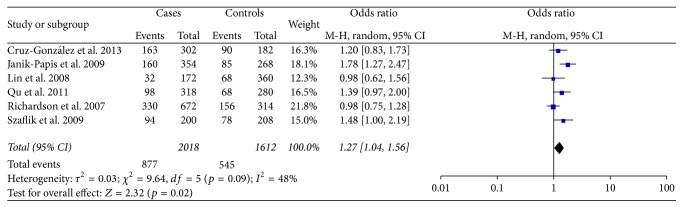
Forest plot of the association between rs833061 polymorphism and wet AMD under an allelic model (C versus T).

Subgroup analysis by subtypes of AMD confirmed that this polymorphism was associated with wet AMD under a homozygote model (CC versus TT: OR = 1.48, 95% CI 1.07–2.04) (Figure 2); a dominant model (CT + CC versus TT: OR = 1.58, 95% CI 1.00–2.51) (Figure 3); and an allelic model (C versus T: OR = 1.27, 95% CI 1.04–1.56) (Figure 4). However, there was no association between this polymorphism and the risk of wet AMD in any of the other genetic models.

**Figure 5:**
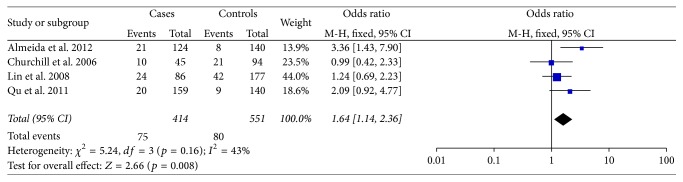
Forest plot of the association between rs1413711 polymorphism and wet AMD under a recessive model (TT versus CC + CT).

For the rs1413711 polymorphism, the meta-analysis showed no significant association between the polymorphism and AMD under a homozygote model (TT versus CC: OR = 1.50, 95% CI 0.71–3.16); a dominant model (CT + TT versus CC: OR = 0.98, 95% CI 0.65–1.49); a heterozygote model (CT versus CC: OR = 0.98, 95% CI 0.65–1.49); a recessive model (TT versus CC + CT: OR = 1.69, 95% CI 0.98–2.89); and an allelic model (T versus C: OR = 1.15, 95% CI 0.86–1.56). However, the stratified analysis indicated that this polymorphism was associated with wet AMD under a recessive model (TT versus CC + CT: OR = 1.64, 95% CI 1.14–2.36) (Figure 5). Subgroup analysis by ethnicity showed no significant association between the polymorphism and AMD in the Asian populations; the stratified analysis was not performed for the Caucasians because only the study by Churchill et al. [19] reported genotype data of a Caucasian population.

For the rs3025039 polymorphism, the meta-analysis showed that no significant association between the polymorphism and AMD was assessed under a homozygote model (TT versus CC: OR = 1.39, 95% CI 0.71–2.73); a dominant model (CT + TT versus CC: OR = 1.07, 95% CI 0.91–1.27); a heterozygote model (CT versus CC: OR = 1.05, 95% CI 0.89–1.25); a recessive model (TT versus CC + CT: OR = 1.39, 95% CI 0.71–2.71); and an allelic model (T versus C: OR = 1.10, 95% CI 0.96–1.27). Further subgroup analyses, by subtypes of AMD and ethnicity, confirmed that there was no association between this polymorphism and the risk of AMD in any of the genetic models.

For the rs2010963 polymorphism, the meta-analysis showed that no significant association between the polymorphism and AMD was assessed under a homozygote model (CC versus GG: OR = 0.81, 95% CI 0.49–1.32); a recessive model (CG + GG versus CC: OR = 0.88, 95% CI 0.58–1.35); a heterozygote model (CG versus GG: OR = 0.85, 95% CI 0.62–1.17); a dominant model (GG versus CC + CG: OR = 0.85, 95% CI 0.63–1.16); and an allelic model (C versus G: OR = 0.96, 95% CI 0.73–1.11). Further subgroup analyses, by subtypes of AMD and ethnicity, confirmed that there was no association between this polymorphism and the risk of AMD in any of the genetic models.

### 3.3. Heterogeneity Across Studies and Sensitivity Analysis

The leave-one-out sensitivity analysis was performed in order to investigate the sources of heterogeneity across studies.

For the rs833061 polymorphism, the *Q*-test and *I* ^2^ statistics showed no significant heterogeneity across studies under the homozygote and recessive models (*p* values > 0.1). Conversely, significant heterogeneity across studies was reported under the heterozygote (*p* < 0.001; *I* ^2^ = 83%), dominant (*p* < 0.001; *I* ^2^ = 82%), and allelic (*p* = 0.09; *I* ^2^ = 47%) models. The sensitivity analysis found that the study by Janik-Papis et al. [18] affected the heterogeneity across studies. When this study was omitted, the between-studies heterogeneity decreased under the heterozygote (*p* = 0.02; *I* ^2^ = 67%), dominant (*p* = 0.03; *I* ^2^ = 62%), and allelic (*p* = 0.47; *I* ^2^ = 0%) models. Particularly, a significant association with AMD was confirmed under an allelic model (OR = 1.19, 95% CI 1.02–1.38).

In the stratified analysis of wet AMD, significant heterogeneity across studies was reported under the dominant (*p* < 0.001; *I* ^2^ = 78%) and allelic (*p* = 0.09; *I* ^2^ = 48%) models. When the study by Janik-Papis et al. [18] was omitted, the between-study heterogeneity decreased under the dominant (*p* = 0.03; *I* ^2^ = 64%) and allelic (*p* = 0.34; *I* ^2^ = 11%) models. However, no significant association was confirmed between the rs833061 polymorphism and wet AMD under both genetic models.

In the subgroup of Caucasians, the *Q*-test and *I* ^2^ statistics showed a significant heterogeneity across studies under the allelic model (*p* = 0.03; *I* ^2^ = 67%). When the study by Janik-Papis et al. [18] study was omitted, the between-studies heterogeneity decreased to *I* ^2^ = 24% (*p* = 0.27) but no significant association was confirmed between the rs833061 polymorphism and AMD, in Caucasians, under the allelic model (OR = 1.16, 95% CI 0.94–1.44). Considering both populations, no significant heterogeneity across studies was reported in any of the other genetic models (*p* values > 0.1).

For the rs1413711 polymorphism, no significant heterogeneity across studies was reported under the heterozygote model (*p* = 0.13). Conversely, significant heterogeneity across studies was reported under the homozygote (*p* = 0.01; *I* ^2^ = 71%), dominant (*p* = 0.06; *I* ^2^ = 60%), recessive (*p* = 0.08; *I* ^2^ = 55%), and allelic (*p* = 0.05; *I* ^2^ = 62%) models. The sensitivity analysis found that the study by Almeida et al. [29] affected the heterogeneity across studies under the homozygote and recessive models. When this study was omitted, the between-study heterogeneity decreased under the homozygote (*p* = 0.10; *I* ^2^ = 57%) and recessive (*p* = 0.43; *I* ^2^ = 0%) models. With regard to the dominant and allelic models, the sensitivity analysis found that the study by Churchill et al. [19] affected the heterogeneity across studies. When this study was omitted, the between-study heterogeneity decreased under the dominant (*p* = 0.47; *I* ^2^ = 0%) and allelic (*p* = 0.20; *I* ^2^ = 38%) models. Particularly, a significant association with AMD was confirmed under the allelic model (OR = 1.29, 95% CI 1.01–1.64).

In the stratified analysis of wet AMD, significant heterogeneity across studies was reported under the homozygote (*p* = 0.02; *I* ^2^ = 68%), dominant (*p* = 0.05; *I* ^2^ = 61%), and allelic (*p* = 0.06; *I* ^2^ = 59%) models. When the study by Churchill et al. [19] was omitted, the between-study heterogeneity decreased under homozygote (*p* = 0.08; *I* ^2^ = 59%), dominant (*p* = 0.39; *I* ^2^ = 0%), and allelic (*p* = 0.26; *I* ^2^ = 26%) models. However, no significant association was reported between the rs833061 polymorphism and wet AMD under these genetic models.

In the subgroup analysis by ethnicity, no significant heterogeneity across studies was reported in any of the genetic models (*p* values > 0.1).

For the rs3025039 polymorphism, the *Q*-test showed no significant heterogeneity across studies under the heterozygote, dominant, recessive, and allelic models (*p* values > 0.1). Conversely, significant heterogeneity across studies was reported under the homozygote model (*p* = 0.06; *I* ^2^ = 56%). The sensitivity analysis found that the study by Tian et al. [33] affected the heterogeneity across studies. When this study was omitted, the between-study heterogeneity decreased (*p* = 0.31; *I* ^2^ = 16%) and a significant association with AMD was showed under the homozygote model (OR = 1.92, 95% CI 1.14–3.22).

In the stratified analysis of wet AMD, significant heterogeneity across studies was reported under the homozygote (*p* = 0.004; *I* ^2^ = 78%), heterozygote (*p* = 0.06; *I* ^2^ = 57%), recessive (*p* = 0.007; *I* ^2^ = 75%), dominant (*p* = 0.01; *I* ^2^ = 69%), and allelic (*p* = 0.002; *I* ^2^ = 77%) models. Particularly, when the study by Tian et al. [33] was omitted, under the recessive model, the between-studies heterogeneity decreased (*p* = 0.17; *I* ^2^ = 44%) and a significant association was reported between the rs833061 polymorphism and exudative form of AMD (OR = 2.49, 95% CI 1.11–5.60).

In the subgroup analysis by ethnicity, no significant heterogeneity across studies was reported in any of the genetic models (*p* values > 0.1).

For the rs2010963 polymorphism, the *Q*-test showed no significant heterogeneity across studies under the homozygote, heterozygote, dominant, and allelic models (*p* values > 0.1). Conversely, significant heterogeneity across studies was reported under the recessive model (*p* = 0.06; *I* ^2^ = 63%). The sensitivity analysis found that the study by Lin et al. [20] affected the heterogeneity across studies. When this study was omitted, the between-study heterogeneity decreased (*p* = 0.50; *I* ^2^ = 0%) but no significant association with AMD was showed under the recessive model.

In the subgroup analyses by subtypes of AMD and ethnicity, no significant heterogeneity across studies was reported in any of the genetic models (*p* values > 0.1).

### 3.4. Publication Bias

The funnel plots of the pooled analyses were quite symmetric. Begg's rank correlation method and Egger's weighted regression method showed that no obvious publication bias for these polymorphisms was found (data not shown).

## 4. Discussion

VEGF is a naturally occurring growth factor selective for endothelial cells that regulates angiogenesis and vascular permeability and plays a leading role in the retinal tissue of AMD, particularly wet AMD. In developed countries, this exudative type of AMD, characterized by the formation of subretinal choroidal neovascularization (CNV), is the major cause of severe vision loss and blindness in the elderly [42, 43]. The association between CNV and increased VEGF expression provides a strong reason for exploring* VEGF* polymorphisms that can contribute to the risk of AMD.

Results by several published studies, evaluating the role of* VEGF* polymorphisms in the AMD development, remain ambiguous, probably due to differences in patient selection, sample size, and genetic and environmental factors. These conflicting findings were summarized by three previous meta-analyses [21–23]. The first published meta-analysis by Lu et al. did not indicate a significant association between rs833061, rs1413711, and rs2010963 polymorphisms and the risk of AMD. However, a subgroup analysis showed significant associations among Caucasian population for rs833061 and rs1413711 polymorphisms and among Asian population for rs1413711 polymorphism [21]. Further, Huang et al. have suggested that the C allele and the CC genotype of rs833061 and the TT genotype of rs1413711 are associated with an increased risk of AMD; particularly, the C allele of rs833061 and the TT genotype of rs1413711 are significant risk factor in the exudative form of AMD. On the contrary, no associations with AMD risk were reported for rs2010963 and rs3025039 polymorphisms [22]. Finally, results by Liu et al. confirmed that the* VEGF* polymorphisms are associated with increased or decreased risk of AMD, particularly wet AMD [23].

Our study, critically reviewing twelve studies, reports results of a more comprehensive meta-analysis and provides a stratified analysis for the exudative form of AMD. The results, by different genetic models, suggested the overall effects of* VEGF* polymorphisms on risk of AMD.

The T allele of the* VEGF* −460 T/C polymorphism (rs833061), located in the promoter region, is associated with a decrease in the promoter activity of the gene [44]. The present meta-analysis showed that subjects who had the C allele had an increased risk of AMD under the homozygote dominant and allelic models. However, the sensitivity analysis, omitting the study by Janik-Papis et al. [18], did not confirm this association under the dominant model. Subgroup analysis by ethnicity confirmed that the Asian carriers of the C allele had an increased risk of AMD under the homozygote, recessive, and allelic models; in Caucasians, the polymorphism was associated with AMD under the homozygote and allelic models. However, when the study by Janik-Papis et al. [18] was omitted, the association under the allelic model was not confirmed in the Caucasian population.

Results by the stratified analysis for wet AMD reported that C allele carriers had an increased risk of the exudative form of AMD under the homozygote, dominant, and allelic models. Though, these findings, under the dominant and the allelic models, were not confirmed by the sensitivity analysis.

For the +674C/T polymorphism (rs1413711), located in intron 1 of the VEGF gene, the meta-analysis did not show an association with the overall AMD risk in any of the genetic models. The sensitivity analysis found that the study by Janik-Papis et al. [18] was a source of heterogeneity across studies. Accordingly, omitting this study from the meta-analysis, a significant association with AMD was confirmed under an allelic model. Furthermore, the stratified analysis indicated that subjects carrying the TT genotype had an increased risk of wet AMD under a recessive model. At the best of our knowledge, no evidence are available regarding the role in the functional activity of this polymorphism, and further analyses are needed in order to clarify the effects of rs1413711 polymorphism on gene expression and protein activities.

Although the meta-analysis did not show an association between the +405G/C polymorphism (rs2010963) and the risk of AMD, the study by Tian et al. [33] was identified as a source of heterogeneity between studies. When this study was omitted from the meta-analysis, a significant association with overall AMD was showed under a homozygote model; particularly, the CC genotype carriers had an increased risk of the exudative AMD.

With regard to the +936C/T(rs3025039) polymorphism, the present study indicated that it is not a risk factor for both overall AMD and wet AMD, in any of the genetic models.

The present study has some limitations. First, the number of studies included in the meta-analysis is modest and some relevant articles were excluded due to insufficient data. Accordingly, the polymorphisms rs833061 and rs3025039 were analysed in six studies, the polymorphism rs1413711 was analyzed in four studies, and the polymorphism rs2010963 was analyzed in three studies. Second, the heterogeneity across studies, which existed for some polymorphisms, must be considered and, although the random effects model and the sensitivity analysis were appropriately performed, the pooled estimates should be interpreted with caution. Finally, since AMD is a complex disorder with sociodemographic, environmental, and genetic risk factors [3, 5], adjusted analyses should be performed, taking into account the confounding factors and gene-environment interactions.

## 5. Conclusion

The present systematic review and the updated meta-analysis constitute a useful guide for readers to study AMD and add new evidence to the growing literature on the role of four common* VEGF* polymorphisms in the risk of AMD. Significant associations with AMD risk were showed for rs833061, rs1413711, and rs3025039* VEGF* polymorphisms but not for rs2010963. However, given the above-mentioned limitations, further studies are needed to better clarify the effect of genetic susceptibility in the development of AMD.

## Figures and Tables

**Table 1:** Characteristics of the studies included in the meta-analysis.

Polymorphism	First author, year	Country	Ethnicity	Type of AMD	Genotype frequency in cases				Genotype frequency in controls				HWE^a^
					Study size	11	12	22	Study size	11	12	22	
rs833061	Richardson, 2007 [25]	Australia	Caucasian	Total AMD	566	154	259	153	157	40	78	39	0.937
				Dry AMD	100	27	43	30	157	40	78	39	
				Wet AMD	336	92	158	86	157	40	78	39	
	Lin, 2008 [20]	China	Asian	Total AMD	190	116	66	8	180	116	60	4	0.239
				Dry AMD	104	60	38	6	180	116	60	4	
				Wet AMD	86	56	28	2	180	116	60	4	
	Janik-Papis, 2009 [18]	Poland	Caucasian	Total AMD	265	48	191	26	134	60	63	11	0.323
				Dry AMD	88	13	67	8	134	60	63	11	
				Wet AMD	177	35	124	18	134	60	63	11	
	Szaflik, 2009 [26]	Poland	Caucasian	Wet AMD	100	14	78	8	104	37	56	11	0.129
	Qu, 2011 [27]	China	Asian	Wet AMD	159	81	58	20	140	81	50	9	0.733
	Cruz-González, 2013 [28]	Spain	Caucasian	Wet AMD	151	31	77	43	91	24	44	23	0.754

rs1413711	Almeida, 2012 [29]	Brazil	Mixed	Total AMD	160	65	66	29	140	67	65	8	0.127
				Dry AMD	36	14	14	8	140	67	65	8	
				Wet AMD	124	51	52	21	140	67	65	8	
	Churchill, 2006 [19]	UK	Caucasian	Wet AMD	45	17	18	10	94	19	54	21	0.147
	Lin, 2008 [20]	China	Asian	Total AMD	190	57	80	53	180	50	85	42	0.617
				Dry AMD	104	29	46	29	180	50	85	42	
				Wet AMD	86	28	34	24	180	50	85	42	
	Qu, 2011 [27]	China	Asian	Wet AMD	159	81	58	20	140	81	50	9	0.733

rs3025039	Galan, 2010 [30]	Finland	Caucasian	Total AMD	226	175	48	3	248	190	54	4	0.942
	Qu, 2011 [27]	China	Asian	Wet AMD	159	114	33	12	140	92	40	8	0.205
	Jiang, 2013 [31]	China	Asian	Total AMD	200	132	50	18	200	138	55	7	0.603
				Dry AMD	49	32	13	4	200	138	55	7	
				Wet AMD	99	66	25	8	200	138	55	7	
	Gonçalves, 2015 [32]	Brazil	Mixed	Wet AMD	88	69	19	0	95	76	19	0	0.279
	Tian, 2012 [33]	China	Asian	Total AMD	533	341	179	13	463	302	143	18	0.835
				Wet AMD	462	299	153	10	463	302	143	18	
	Lin, 2008 [20]	China	Asian	Total AMD	190	120	58	12	180	134	42	4	0.742
				Dry AMD	104	75	27	2	180	134	42	4	
				Wet AMD	86	45	31	10	180	134	42	4	

rs2010963	Lin, 2008 [20]	China	Asian	Total AMD	190	40	132	18	180	34	116	30	<0.001
				Dry AMD	104	24	70	10	180	34	116	30	
				Wet AMD	86	16	62	8	180	34	116	30	
	Janik-Papis, 2009 [18]	Poland	Caucasian	Total AMD	265	164	84	17	134	85	44	5	0.813
				Dry AMD	88	47	30	11	134	85	44	5	
	Qu, 2011 [27]	China	Asian	Wet AMD	159	54	70	35	140	39	74	27	0.442

__^a^ *p* value for Hardy Weinberg equilibrium in controls.
